# Erector spinae plane block for affective and safe analgesia in a patient with severe penetrating chest trauma caused by an explosion in the battlefield

**DOI:** 10.1002/ccr3.6433

**Published:** 2022-10-08

**Authors:** Dmytro Dmytriiev, Dan Sebastian Dîrzu, Mykola Melnychenko, Rudiger Eichholz

**Affiliations:** ^1^ Anestesia and Intensive Care Department Vinnytsya National Pirogov Memorial Medical University Vinnytsia Ukraine; ^2^ Anestesia and Intensive Care Department Clinicilor 4‐6 Emergency County Hospital Cluj‐Napoca Romania; ^3^ medizi. Private Practice for Anaesthesiology Stuttgart Germany

**Keywords:** combat anesthesia, continuous peripheral nerve block, erector spinae plane block (ESP), postoperative regional anesthesia

## Abstract

The ongoing conflict in Ukraine continues to generate many complex traumatic injuries and provides unique challenges to anaesthesiologists who provide medical care at various levels of medical evacuation. We report the successful use of an ultrasound‐guided continuous erector spinae plane (ESP) block in a patient with severe posterolateral chest trauma. The acute perioperative outcome of the patient was improved with the ESP block, the main benefits being excellent analgesia and minimal postoperative morphine requirements without influencing the risk of bleeding and coagulopathy. We conclude that continuous ESP block can be utilized to provide excellent analgesia following massive thoracic trauma. It's ease of placement under ultrasound guidance and low risk of complications makes this technique particularly useful in war medicine.

## Graphical abstract

Erector spinae block is effective and safe in treating severe chest pain caused by penetrating trauma after explosion in war setup.

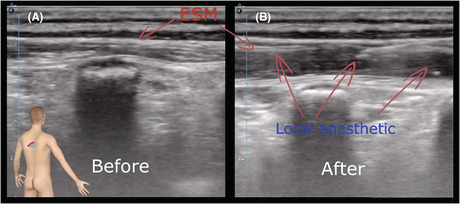

## 1 INTRODUCTION

The military conflict in Ukraine continues to generate many complex multi‐traumas among the military and civilians. This creates new challenges for anaesthesiologists who provide medical care at various levels of medical evacuation. Penetrating injuries due to explosive devices are a common cause of chest injuries in the current combat environment. Chest injuries are more common in the military than in civilians.^1^ After the immediate care such as chest drainage in case of pneumothorax, thoracic trauma is characterized by extremely severe and prolonged pain because of the physiological movements of the chest.^2^, ^3^ Acute pain limits the patient's ability to breathe deeply and promotes shallow breathing, leading to hypoxemia, atelectasis, and, in some cases, pneumonia.^1^, ^2^, ^3^ Adequate analgesia is the keystone of trauma management and it has a role in preventing the development of the above described secondary effects and complications. However, morphine consumption may be detrimental, as increased doses, without being fully effective in alleviating the pain, can sedate the patient and decreases the respiratory drive. On a long‐term, avoiding opioid in the acute settings may decrease the risk of chronic misuse of these drugs in the future, with an positive overall impact on the quality of live of our patients. It was shown that opioids misuse in veterans is associated with severe negative side effects like greater depression, anger, sleep disturbance, AUDIT scores, PTSD symptoms, suicidality, and pain interference .^4^

It is expected that the use of regional techniques, by providing excellent analgesia and decreasing morphine consumption, will increase treatment outcomes and patient safety. Currently, there is a significant range of possible blockades of peripheral nerve plexuses and fascial plane blocks for analgesia. Our experience has shown that these methods are effective and contribute to the rapid mobilization of patients, and thus, contribute significantly to the recovery of war victims. In this report, we share our experience of using an erector spinae plane (ESP) block under ultrasound guidance for perioperative analgesia in a soldier with severe chest combat trauma.

## 2 CASE PRESENTATION

A 37‐year‐old man was evacuated by helicopter from the combat zone to a military hospital 2 h after suffering a chest injury secondary to an explosion. The patient was air‐lifted by the country's medical forces and received first aid on the way: opioid analgesia, aseptic dressing, upright positioning, and oxygen therapy. Upon arrival, the patient was agitated and complained of respiratory distress and chest pain that worsened with movement. Examination revealed a penetrating chest wound on the right posterolateral side, covered with an occlusive dressing. No penetrating abdominal injuries were detected. Auscultation of the lungs revealed a significant weakening of respiration on the side of the injury. An available portable ultrasound machine with a high‐frequency linear sensor (3–16 MHz) was used to confirm the suspicion of pneumothorax. The ultrasound sensor was placed in a parasternal position on the upper part of the chest between the second and third ribs.

**FIGURE 1:**
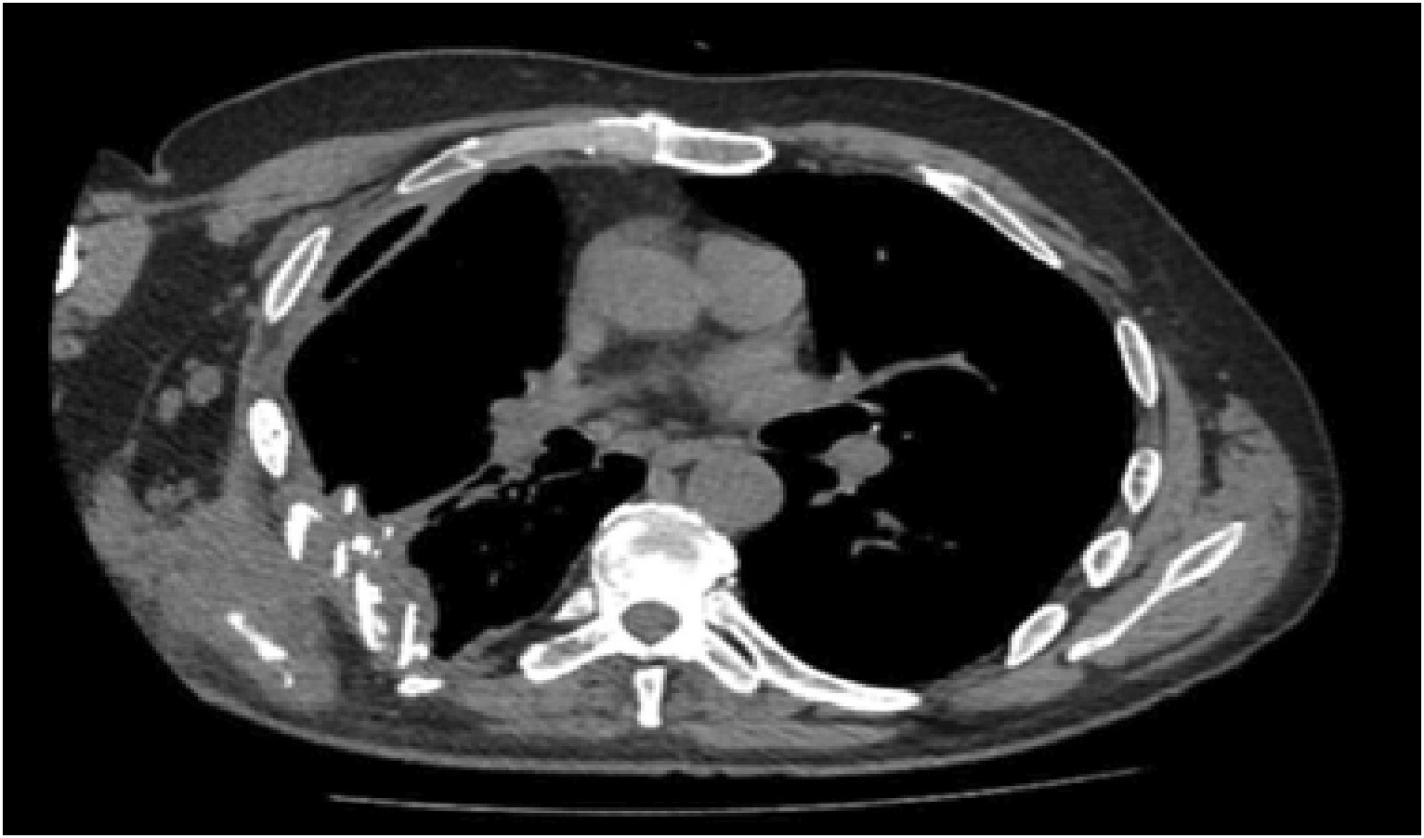
MRI scan showing chest wall deformity and rib fractures

**FIGURE 2:**
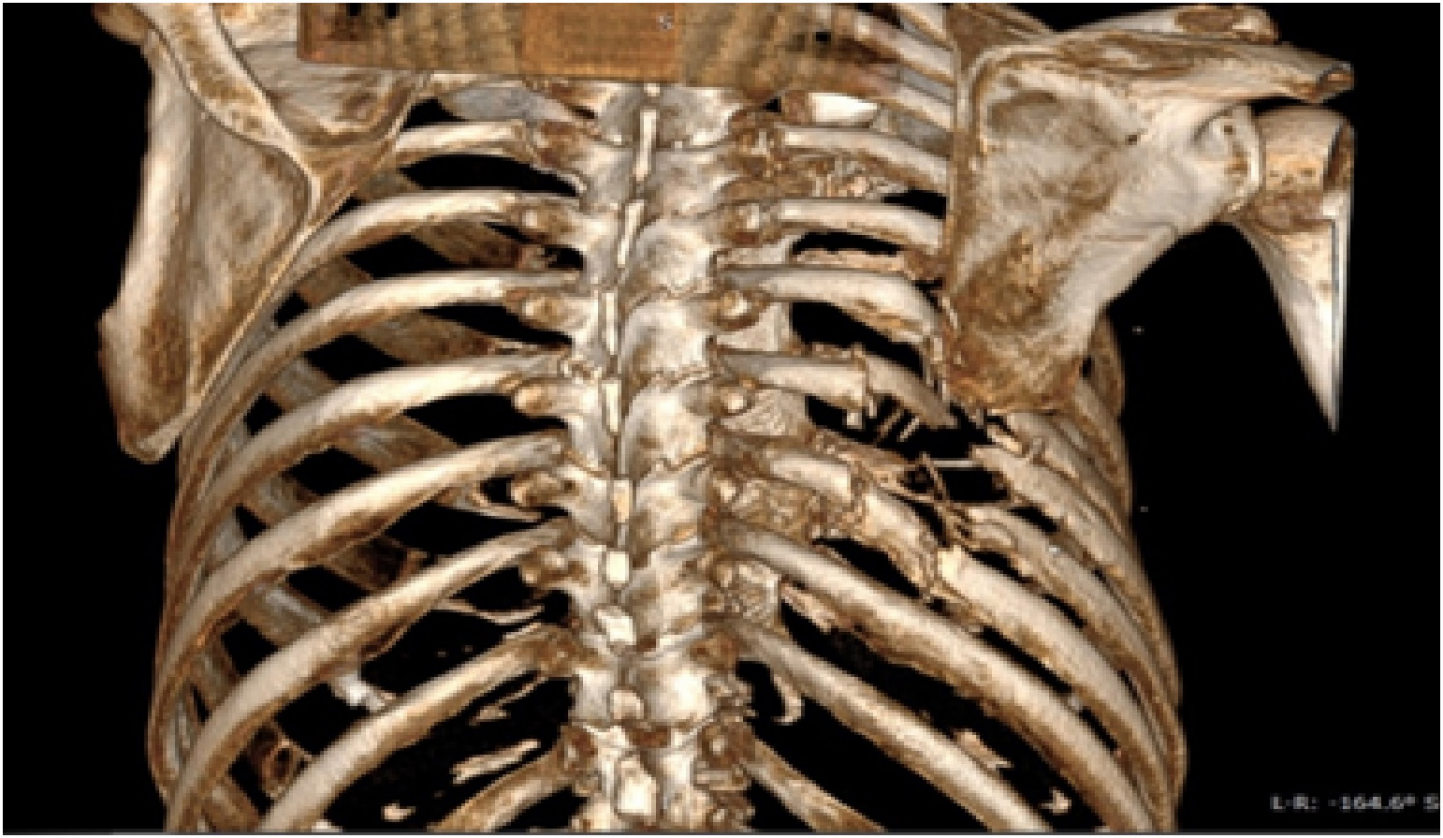
3D CT examination showing the extension of rib fractures.

Based on MRI and CT with 3D imaging (Figures 1 and 2), damage of the other organs was ruled out and the final diagnosis was made: multiple fractures of the ribs on the right (Th IV‐IX), trauma of the right lung with an explosion fragment retained inside the thoracic cavity.

The patient underwent puncture and drainage of the pleural cavity on the right side with free evacuation of the air. The patient complained of significant pain in the right side of the chest, despite repeated doses of fentanyl and ketamine. To facilitate pain control in the perioperative period, it was decided to perform an ultrasound‐guided ESP block with catheter insertion. The purpose was to improve respiratory function and patient comfort by offering regional pain relief and reduction of opioid consumption with subsequent lesser central breathing depression.

**FIGURE 3:**
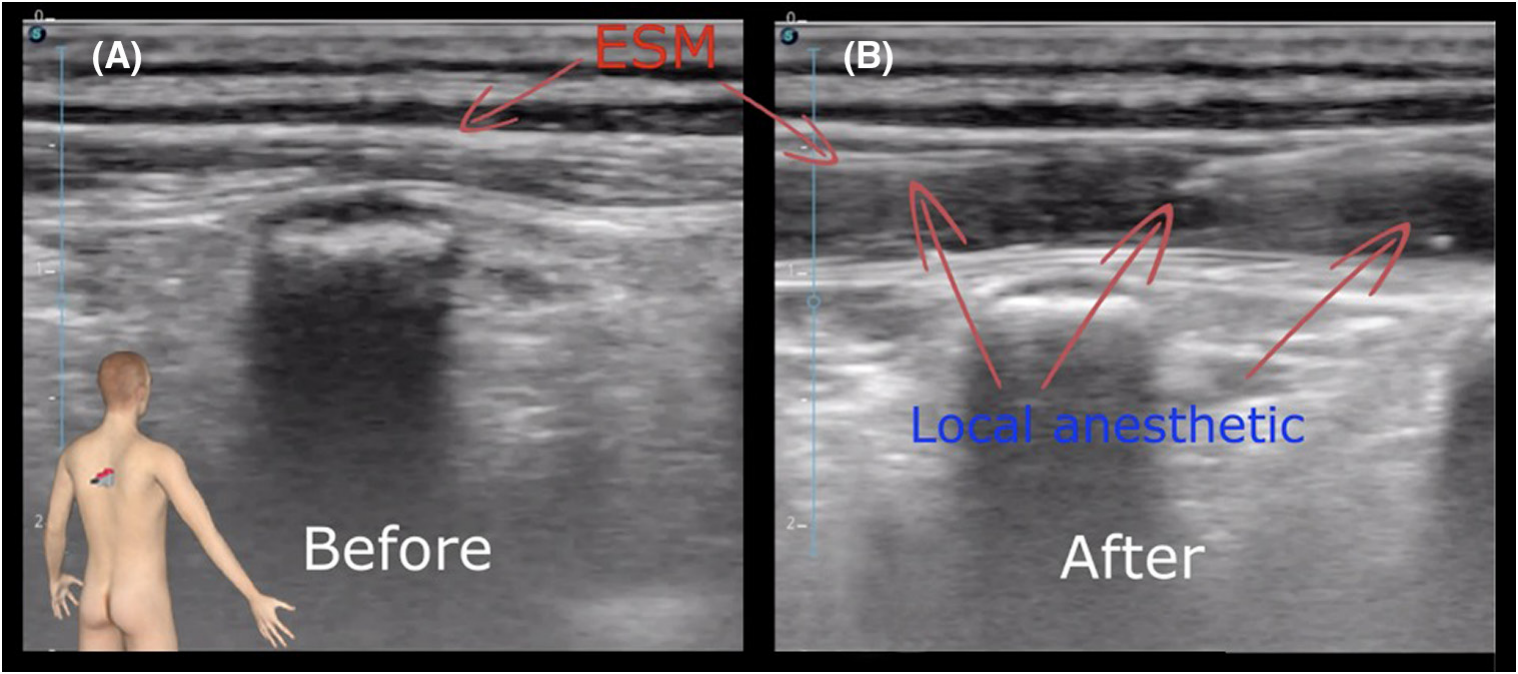
Ultrasound‐guided Erector Spinae Plane Block using a high‐frequency linear transducer (3–16 MHz) ((A): before injection, (B): after injection, ESM: Erector spinae muscles).

The patient was prepared for regional anesthesia in the operation room in a sitting position with standard monitoring of the vital functions. Sterile conditions were established. Injection sites at the Th5, Th6, and Th7 levels were identified using a high‐frequency (3–16 MHz) linear probe. We performed a multi‐injection‐technique at the three levels mentioned above to ensure a good local anesthetic spread. The block was performed under constant in‐plane needle visualization. To prevent intravascular injection, aspiration was performed at each level and then 10 ml of 0.5% Bupivacaine was administered. The correct placement was confirmed by the lateral distribution of the local anesthetic and the separation of the erector spinae muscles from the transverse process (Figure 3). The catheter was placed at the level of Th6. Subcutaneous tunneling was performed for catheter fixation, and a transparent sterile dressing was applied.

Assessment of pain was performed using a visual analog scale (VAS). The scale is graduated from 0 to 10, where 0 means no pain and 10 is the most severe pain the patient can imagine. Within a few minutes after injection, the patient reported a significant drop of his pain level from VAS 7 to VAS 3. There were no clinical signs for complications after the procedure.

Thoracotomy was performed under general anesthesia to remove the retained fragment and for lung suture. After surgery, a pump for continuous infusion of local anesthetic was connected to the catheter. A continuous infusion of 0.2% Bupivacaine at a rate of 6 ml/h through an elastomeric pump was started.

Indicators of vital functions in the postoperative period are presented in Table 1.

**TABLE 1:** Hemodynamic parameters and ventilation status of the patient in postoperative period

Time after surgery	6 h	12 h	24 h	48 h	72 h	96 h	120 h
Patient gas exchange							
PaO_2_ (mmHg)	68 72 76			80	82	85	87
PaCO_2_ (mmHg)	49	47	43	40	39	37	37
SpO_2_ (%)	91	93	94	94	96	97	98
Hemodynamic parameters							
BP (mmHg) MAP (mmHg)	145/90 108.3	140/85 103.3	140/85 103.3	130/80 96.7	125/80 95	120/80 93.3	120/80 93.3
HR (beats/min)	107	104	98	92	86	82	84

On the 2nd day, the intensity of pain at rest was VAS 1–2, with movements and mobilization 3 points. The infusion of local anesthetic was continued uninterrupted for 7 days, after which the catheter was removed. The dynamics of the pain syndrome and the need for opioid analgesia are presented in Table 2.

**TABLE 2:** The dynamics of the pain syndrome and the need for opioid analgesia

Time after surgery	6 h	12 h	24 h	48 h	72 h	96 h	120 h
VAS (1–10)	5	3	3	2	2	1	1
The number of injections of morphine	3	2	2	0	0	0	0

Toxic effects of local anesthetics or other complications were not detected during the 7 days of analgesic treatment with the ESP catheter.

## 3 DISCUSSION

During transportation, opioid analgesia was provided, but its subsequent use did not reduce the pain to a tolerable level while significant signs of respiratory depression was present. The multimodal analgesic approach in our case was provided with an ESP block to reduce acute pain and prevent the development of probable secondary adverse outcomes. Several methods of analgesia for the treatment of chest injuries have been described in the literature. Epidural anesthesia has been described as the most effective technique,^3^ but combat trauma is often accompanied by bleeding and the need for blood transfusions with subsequent possible coagulopathy. Based on the data from the Second Consensus Conference of the American Society for Regional Anesthesia and Pain Medicine on neuraxial anesthesia and anticoagulation, the use of epidural anesthesia is limited in our case due to the likelihood of developing epidural hematoma with subsequent neurological dysfunction.^5^ The same applies for the paravertebral block. Positioning the patient with combat wounds for an epidural can be challenging and sometimes even impossible due to the complexity of the lesions. As an alternative to neuraxial anesthesia, the ESP block was chosen. ESP is a relatively new interfascial blockade able to provide somatic and visceral analgesia. The exact mechanism of action is yet to be established, but it is supposed to be blocking mostly the dorsal branches of the spinal nerve. The wide craniocaudal distribution of the anesthetic is useful for blocking many dermatomes.^6^ ESP block has already been described as an effective method of treating chest neuropathic pain and acute pain after thoracotomy.^7^ The blockade is not technically difficult if there is a clear ultrasound image and does not require patient to assume difficult position. Compared with other chest blockades, there is a low risk of complications such as pneumothorax, bleeding, or hematoma formation.^8^ A multimodal approach to pain relief in combat injuries using regional techniques seems to be an important element of effective and safe pain control.

## 4 CONCLUSIONS

We describe the successful use of the ESP block in a patient with a severe chest injury secondary to an explosion in combat zone. The ESP block proved to be both effective and safe offering excellent analgesia with no side effects, decreasing morphine consumption, and facilitating recovery. By using ESP block, not only we provided effective analgesia, but also we were able to early discharge the patient from the hospital with good respiratory function. The disadvantages of central neuraxial analgesia in the presence of coagulopathy or other contraindications were avoided.

## AUTHOR CONTRIBUTIONS

DD and MM were involved in treating the patient, documenting the case, and writing the draft. DDS and RE reviewed the manuscript. All the authors were involved in designing the report.

## CONFLICT OF INTEREST

DDS has SE role in the CCRJ editorial team. No other COI to declare.

## CONSENT

Written informed consent was obtained from the patient to publish this report in accordance with the journal's patient consent policy.

## Data Availability

Data sharing not applicable to this article as no datasets were generated or analysed during the current study.
